# Interplay of autophagy and Th1/Th2-mediated macrophage polarization in host-pathogen dynamics

**DOI:** 10.3389/fcimb.2025.1679514

**Published:** 2025-10-02

**Authors:** Gaurav Shoeran, Namrata Anand

**Affiliations:** ^1^ Department of Pharmaceutical Sciences, College of Pharmacy, University of Kentucky, Lexington, KY, United States; ^2^ Section of Hematology and Oncology, College of Medicine, University of Chicago, Chicago, IL, United States

**Keywords:** M1/M2 macrophage polarization, autophagy, host-parasite interaction, immune response, Th1/Th2 response, Mycobacterium, Salmonella, *Toxoplasma gondii*

## Abstract

Autophagy, host immune responses, and macrophage polarization form a tightly regulated network. This network significantly influences the outcome of intracellular pathogenic infections. Autophagy acts as a critical cellular defense mechanism. It degrades intracellular pathogens and helps with antigen presentation in antigen presenting cells like macrophages. Intracellular parasites have evolved diverse strategies to modulate autophagy. They may inhibit autophagosome formation, block autophagosome-lysosome fusion, or redirect autophagic flux for their survival. These manipulations allow pathogens to evade degradation and persist within host cells. Macrophage polarization further influences autophagic activity: M1 macrophages typically exhibit enhanced autophagy, supporting antimicrobial functions, while M2 macrophages show reduced autophagic flux, contributing to immune regulation and tissue repair. Autophagy itself can influence macrophage phenotypes, with its activation promoting M1-like characteristics and its inhibition favoring M2-like responses. The macrophage polarization states influence T cell polarization and infection outcome. This bidirectional relationship between autophagy and macrophage polarization plays a pivotal role in determining host resistance or susceptibility to intracellular pathogens. In this review, we highlight findings from macrophage-infecting pathogens that manipulate autophagy, macrophage and T cell to enhance their survival within the host.

## 1 Introduction

Autophagy is a vital cellular process that maintains homeostasis by degrading and recycling damaged organelles, misfolded proteins, and other cellular debris. It is especially important during stress conditions like nutrient deprivation, where it provides energy and building blocks for survival (Mizushima, 2007). The process begins with the formation of a double-membraned structure called the autophagosome, which engulfs the targeted cellular components. This autophagosome then fuses with a lysosome, where its contents are broken down and reused (Tanida, 2011). Autophagy is tightly regulated by signaling pathways, particularly the mTORC1/mTORC2 complex, which inhibits autophagy when nutrients are plentiful, and activates it when resources are scarce. This balance ensures that cells adapt efficiently to changing environmental conditions (Linke et al., 2017).

Autophagy is a cellular degradation process that can be either canonical (non-selective) or selective, depending on the nature of the cargo and the triggering signals. Non selective canonical autophagy begins with signal induction, where nutrient deprivation or specific cargo (e.g., damaged mitochondria or pathogens) activates the ULK1 complex (ULK1, ATG13, FIP200, ATG101) (Lin and Hurley, 2016). Under nutrient-rich conditions, mTORC1 inhibits ULK1, but during starvation or stress, ULK1 is activated and initiates autophagosome formation. ULK1 recruits the Beclin 1 complex (BECN1, ATG14L, PIK3R4), which activates PtdIns3KC3 to produce PtdIns3P, attracting proteins like WIPI1/2 and ATG9 to nucleate the phagophore membrane (Russell et al., 2013). Vesicle expansion involves two ubiquitin-like conjugation systems: ATG12–ATG5–ATG16L1 and LC3–PE, with enzymes like ATG7, ATG10, ATG3, and ATG4B facilitating conjugation and recycling (Russell et al., 2013). Autophagosomes then fuse with lysosomes (or vacuoles in yeast) via SNARE proteins (e.g., syntaxin 17, VAMP8) and GTPases like RAB7, enabling cargo degradation by lysosomal hydrolases (Diao et al., 2024). In selective autophagy, cargo recognition is mediated by receptors/adaptors (e.g., p62, NBR1, NDP52, optineurin) that bind both ubiquitin-tagged cargo and LC3 through LC3-interacting regions (LIRs), ensuring targeted degradation of specific substrates like protein aggregates, peroxisomes, or bacteria (Vargas et al., 2023).

Xenophagy is a specialized form of selective autophagy that targets and eliminates intracellular invaders. Unlike non-selective autophagy, which degrades bulk cytoplasmic material during nutrient stress, xenophagy is triggered by the presence of foreign entities within the cell. Xenophagy is initiated by the ubiquitination of either the pathogen itself or its damaged vacuole. This tagging process involves a cascade of enzymes—E1 (activating), E2 (conjugating), and E3 (ligating)—with E3 ligases like LRSAM1 and Parkin playing key role (Sharma et al., 2018). It relies on the recognition of pathogen-associated molecular patterns and damage signals, often marked by ubiquitination (Sharma et al., 2018). Autophagy receptors such as p62, NDP52, and optineurin bind to these ubiquitin-tagged cargos and link them to LC3 on the forming autophagosome membrane via LIRs (Ryan and Tumbarello, 2018; Bruqi and Strappazzon, 2025). This process recruits autophagic proteins such as ULK1, ATG9L1, ATG16L1, and ATG14L, even in the absence of LC3, indicating that xenophagy can proceed independently of classical autophagy receptors. This ensures precise sequestration of the invader into autophagosomes, which then fuse with lysosomes for degradation. While the goal is to assemble autophagy machinery and degrade the pathogen, many microbes have evolved strategies to evade or exploit this system. Xenophagy plays a crucial role in maintaining cellular integrity and contributes to innate immune defense by preventing pathogen replication and promoting antigen presentation (Wang and Li, 2020).

Alternative or non-canonical autophagy refers to pathways that bypass some components of the classical autophagy machinery. For instance, ULK1/2-independent autophagy can occur during prolonged glucose starvation/ammonia induced (Cheong et al., 2011), and ATG5-independent autophagy—important in erythrocyte maturation—relies on ULK1 and BECN1 but not on LC3–PE conjugation or other core proteins like ATG7, ATG12, ATG16L1, and ATG9 (Cheong et al., 2011). Conversely, BECN1-independent autophagy has been observed in response to apoptotic stimuli and certain toxins, requiring ULK1, ATG5, ATG7, and LC3–PE, though its mechanism remains unclear (Deng et al., 2022). Another form, endosome-mediated autophagy, originates from late endosomes (MIICs) in dendritic cells and is independent of ATG4B and LC3–PE (Kondylis et al., 2013). Additionally, LC3-associated phagocytosis (LAP) (Cemma et al., 2011), which occurs during TLR- or Fc receptor-mediated phagocytosis, involves LC3 and other autophagy proteins but not the ULK1 complex or double-membrane autophagosomes (Schille et al., 2018). LAP depends on NADPH oxidase and ROS production and plays a role in controlling intracellular pathogens (Cheng et al., 2019).

mTORC1 and mTORC2 are two distinct complexes within the mTOR signaling pathway that play crucial roles in cellular growth, metabolism, and immune regulation (Szwed et al., 2021). mTORC1 is primarily activated by amino acids, energy levels, and growth factors, and it promotes anabolic processes like protein synthesis while inhibiting autophagy. It signals through downstream targets such as S6K1 and 4E-BP1 and is sensitive to rapamycin (Szwed et al., 2021). In immunity, mTORC1 supports pro-inflammatory responses, including Th1 and Th17 differentiation (Yurchenko et al., 2012; Liu et al., 2015; Wu et al., 2015; Pandit et al., 2021). In contrast, mTORC2 is activated mainly by growth factors via PI3K signaling and regulates cell survival, cytoskeletal organization, and metabolism through targets like AKT, SGK1, and PKC (Fu and Hall, 2020). It is less sensitive to rapamycin and plays a role in promoting Th2 and regulatory T cell (Treg) responses, contributing to immune balance and tolerance (Chapman and Chi, 2014; Wang et al., 2020; Harvey et al., 2022). Together, these complexes coordinate cellular responses to environmental cues and help fine-tune immune function.

Autophagy plays a pivotal role in regulating macrophage polarization, influencing the balance between pro-inflammatory M1 and anti-inflammatory M2 phenotypes. It functions as a cellular reprogramming mechanism that can shift macrophages between polarization states. (Zubrova and Morshneva, 2024). Macrophage polarization is a dynamic and context-dependent process that reflects the activation state of macrophages at a specific point in space and time. It broadly categorizes macrophages into M1 (classically activated) and M2 (alternatively activated) phenotypes, though these terms are simplifications of a much more complex spectrum. M1 macrophages, induced by signals like IFN-γ and LPS, are pro-inflammatory and antimicrobial, expressing markers such as *Il1β*, *Tnf*, and *Nos2*, and are essential for Th1 responses and pathogen clearance. M2 macrophages, stimulated by IL-4 and IL-13, are involved in tissue repair and immune regulation, marked by genes like *Retnla*, *Arg1a*, *Irf4*, and *Pparg*+. Knockout studies have helped identify key regulators of these phenotypes, though many genes show context-specific or undefined roles, highlighting the complexity of macrophage biology.

**Figure 1:**
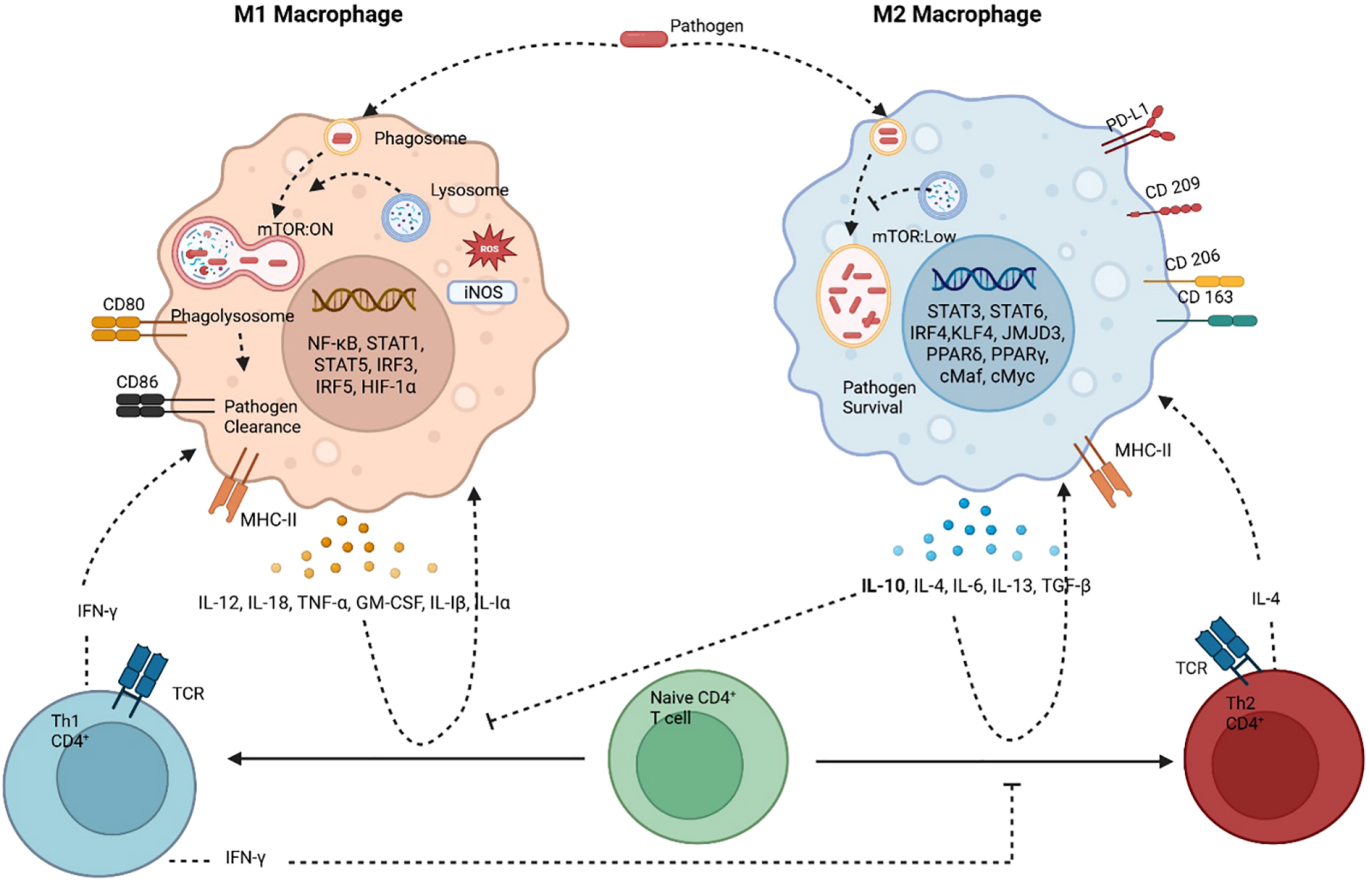
Macrophages and T cells constantly interact with each other through cytokines, antigen presentation, metabolic states and physiological states to determine macrophage polarization and TH1/TH2 skewed T cell response. M1 macrophages (classically activated) have heightened autophagy which aids in pathogen clearance. M1 macrophages are pro-inflammatory, antimicrobial and tumoricidal. The M1 macrophages are associated with TH1 CD4+ T cells, which activate each other by antigen presentation and a crosstalk of cytokines. The other type is the alternatively activated M2 macrophages, this phenotype is associated with autophagy inhibition and pathogen/microbial persistence. It’s anti-inflammatory and associated with tissue repair and is tumor promoting. This phenotype is accompanied by TH2 skewed T cell response. Dominance of one type of response is inhibitory to the other, TH1 cytokine IFN-Υ inhibits TH2 phenotype and TH2 cytokine IL-10 inhibits TH1 phenotype. Created in BioRender. Shoeran, et al. (2025) https://BioRender.com/2in15jf.

Importantly, polarization is not a fixed state but a reflection of macrophage behavior in response to environmental cues, often lacking granularity in traditional M1/M2 classification. The phenotype of a macrophage does not always predict its function, making it essential to link molecular pathways to physiological outcomes. Advances in single-cell technologies are uncovering the heterogeneity and plasticity of macrophage activation, moving the field toward more precise definitions. Polarization is tightly linked to the resolution or persistence of inflammation and pathogenic infections—where resolving inflammation restores tissue homeostasis, and nonresolving inflammation perpetuates disease. Therefore, understanding macrophage polarization and its integration with other cellular pathways-such as autophagy-is essential for unraveling immune responses in infection, cancer, and chronic inflammatory diseases. **Figure 1** illustrates how autophagy modulates macrophage polarization and how these polarization states, in turn, influence T cell differentiation and their interactions.

## 2 AKT-MTORC signaling in autophagy and macrophage polarization

Macrophages are dynamic immune cells that respond to pathogens and cellular debris by initiating and resolving inflammation. Their activation is mediated by signaling cascades downstream of pattern recognition and cytokine receptors, leading to transcriptional and epigenetic changes that drive cytokine production, migration, and pathogen clearance (Mosser, 2003). Macrophage activation states are broadly categorized into M1 and M2 phenotypes, each representing a spectrum of functional responses shaped by specific stimuli and environmental cues. M1 macrophages are typically pro-inflammatory, while M2 macrophages are associated with tissue repair and immune regulation (Italiani and Boraschi, 2014; Anand et al., 2023). M1/M2 macrophage polarization has played an important role in cancer therapy, infectious disease and drug discovery with special emphasis on exosomes/vesicles based cargo therapy (Anand et al., 2023; Cao et al., 2024; Schweer et al., 2023; Schweer et al., 2024). The PI3K/Akt signaling pathway plays a central role in determining macrophage phenotype, integrating signals from various receptors to modulate inflammatory responses and polarization. This pathway promotes M2 activation and anti-inflammatory functions, although specific isoforms can also support M1 responses, highlighting its versatile role in immune regulation in cancer and microbial diseases (Lu et al., 2017; Linton et al., 2019; Wu et al., 2025).

The Akt-mTORC1 axis also integrates metabolic and epigenetic signals to regulate macrophage polarization. IL-4-induced M2 polarization requires Akt-mTORC1-mediated activation of ATP-citrate lyase (Acly), which generates acetyl-CoA for histone acetylation and transcription of M2-specific genes (Covarrubias et al., 2016). However, constitutive activation of mTORC1, such as in Tsc1^Δ/Δ^ macrophages, disrupts this balance. These cells exhibit impaired M2 polarization despite intact STAT6 signaling, due to feedback inhibition of Akt and defective metabolic reprogramming (Huang et al., 2008). This underscores the dual role of Akt: while it supports M2 gene expression and metabolism, its overactivation through mTORC1 can paradoxically suppress these functions.

The PI3K/Akt signaling pathway plays a central role in regulating macrophage survival, migration, and response to metabolic and inflammatory cues. Activated by receptors such as TLRs, cytokine receptors, and Fc receptors, PI3K catalyzes the formation of PIP3, which recruits and activates Akt via mTORC2 (Fukao and Koyasu, 2003). Akt then inhibits the TSC1/2 complex, leading to mTORC1 activation. This pathway modulates cytokine production and acts as a negative regulator of TLR and NF-κB signaling, thereby restricting proinflammatory responses and promoting anti-inflammatory outcomes (Yang et al., 2006; Weichhart et al., 2008; Chaurasia et al., 2010). PI3K/Akt signaling is essential for M2 macrophage polarization, with Akt activation required for the expression of M2-associated genes (Arranz et al., 2012). Signals like IL-4, IL-10, TGF-β, and BMP-7 utilize this pathway to induce M2 phenotypes, while inhibition of PI3K/Akt enhances M1-type responses (Park et al., 2011; Gong et al., 2012; Rocher and Singla, 2013).

**Figure 2:**
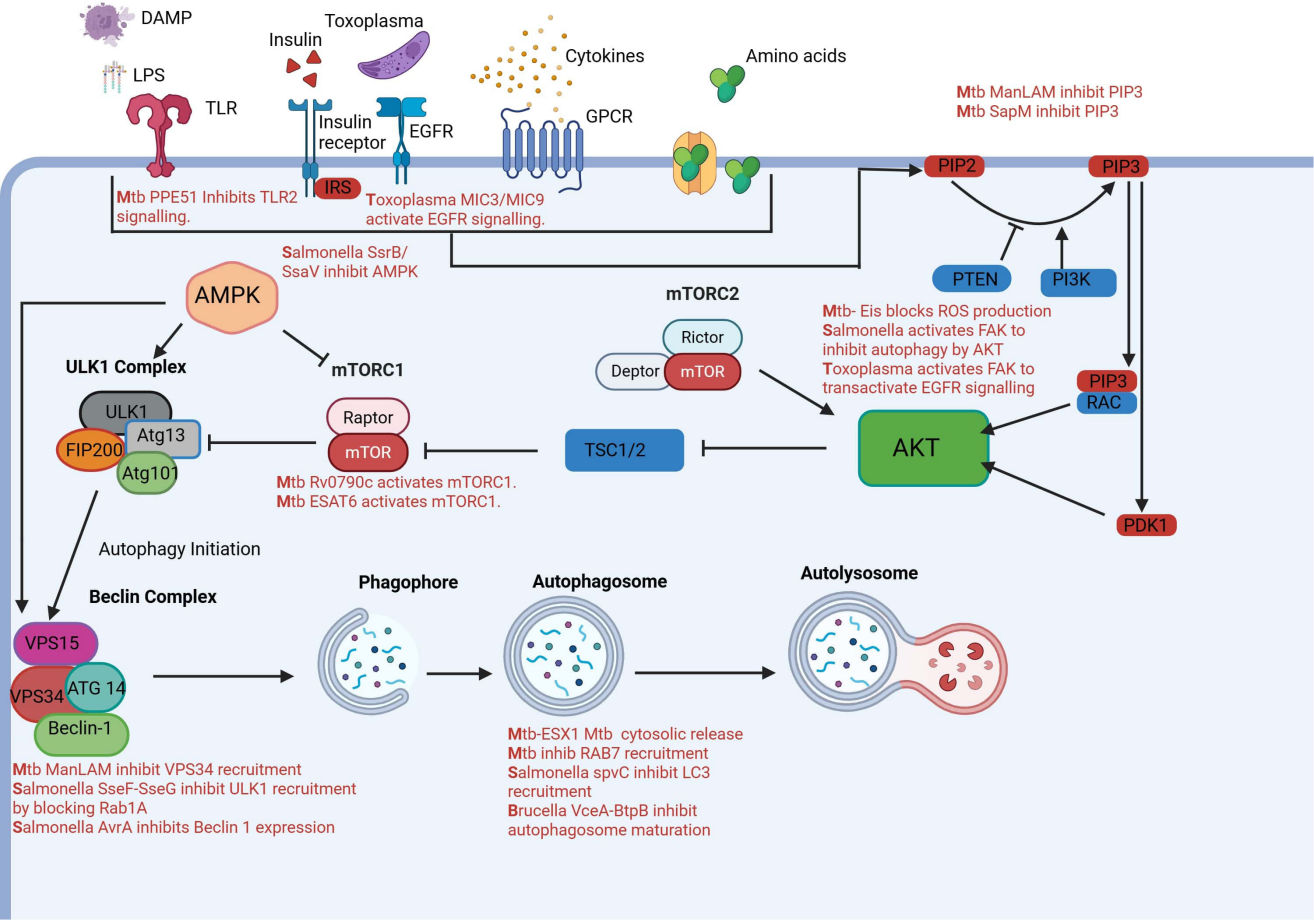
AKT activation in macrophages can be activated by PIP3 and PDK1 in response to extracellular stimuli like amino acids, growth factors, cytokines, LPS, DAMPS and *Toxoplasma gondii* microneme proteins. Once activated AKT inhibits autophagy by inhibiting mTORC1 complex. AKT itself is regulated by mTORC2. AMPK also controls autophagy by sensing nutrients and inhibiting or activating mTORC1.Autophagosome formation is initiated by the ULK1 complex and then BECLIN-1 complex leads to formation of autophagosome which finally fuses with lysosomes to digest intracellular pathogens. Pathogens have acquired several strategies to manipulate this pathway inside of macrophages to aid in their survival. Created in BioRender. Shoeran, et al. (2025) https://BioRender.com/v11t1nw.

Macrophages play a central role in innate immunity by actively migrating to sites of infection or tissue damage and eliminating pathogens through phagocytosis. This process is initiated by chemokine signaling, which promotes actin polymerization and cytoskeletal rearrangement, enabling macrophage movement and engulfment of foreign bodies. Among Akt isoforms, Akt2 is particularly important for chemotaxis and filopodia formation, with its absence impairing chemokine-induced actin remodeling and migration (Zhang et al., 2009). Intracellular pathogens have evolved mechanisms to exploit this signaling axis to their advantage. *Mycobacterium tuberculosis(M.tb)* activates PI3K/Akt/mTORC1 signaling to inhibit autophagy, preventing its degradation within macrophages. It enhances phosphorylation of Akt and mTOR, thereby blocking autophagosome maturation and lysosomal fusion (Deretic, 2008). Similarly, *Salmonella enterica* manipulates host focal adhesion kinase (FAK), which activates Akt and mTORC1 (Shi and Casanova, 2006), suppressing autophagy and promoting bacterial survival within Salmonella-containing vacuoles. Other pathogens also target this axis to modulate macrophage polarization and immune responses, often skewing signaling toward anti-inflammatory or survival-promoting pathways. These strategies allow pathogens to persist within host cells, evade immune clearance, and contribute to chronic infection (Muraille et al., 2014). Thus, the balance between Akt-mediated promotion of phagocytosis and inhibition of autophagy is not only central to macrophage function but also a key target of pathogen manipulation. **Figure 2** illustrates the upstream signaling pathways that regulate autophagy in response to various stimuli. It also highlights how different intracellular pathogens interact with the autophagy machinery, which will be discussed in detail in the following sections.

## 3 Host autophagy and intracellular pathogens

### 3.1 Mycobacterium

Mycobacteria are a group of slow-growing, acid-fast bacteria known for causing diseases like tuberculosis (*M.tb*) and leprosy (*Mycobacterium leprae*). They primarily reside in macrophages, a type of immune cell, where they survive and replicate by evading the host’s immune defenses.

### 3.2 Host autophagy in *Mycobacterium* clearance

Autophagy is a critical host defense mechanism that targets intracellular pathogens like *M.tb* for degradation. This process, particularly xenophagy—a selective form of autophagy—enables macrophages to sequester and eliminate *M.tb* within autophagosomes that fuse with lysosomes. The initiation of autophagy in response to *M.tb* is tightly regulated by pattern recognition receptors (PRRs), especially Toll-like receptors (TLRs), which detect pathogen-associated molecular patterns (PAMPs) and activate downstream signaling cascades, including those involving MyD88 and TRIF (Kleinnijenhuis, et al., 2011a, b; Liu et al., 2017; Sepehri et al., 2019). These pathways not only trigger inflammatory responses but also promote autophagic flux, enhancing the host’s ability to control infection.

Recent studies have highlighted the role of membrane stress responses, particularly Atg8ylation, in mediating autophagy during *M.tb* infection. Atg8ylation involves the conjugation of ATG8-family proteins (e.g., LC3) to damaged or stressed membranes, serving as a broader cellular response to membrane perturbation (Chen et al., 2025). This process is essential for both canonical autophagy and non-canonical pathways like LAP, which are activated during *M.tb* infection (Deretic and Wang, 2023). These mechanisms contribute to bacterial clearance and modulation of inflammation, although their effectiveness can vary depending on the host cell type and infection context.

Dynamic imaging studies have revealed that LC3 recruitment to *M.tb*-containing vacuoles (MCVs) is highly variable and does not always lead to successful autophagosome maturation or acidification. This suggests that while autophagy is activated, its bactericidal efficacy may be limited or transient in human macrophages (Okugbeni et al., 2022). Moreover, the recruitment of LC3 is often associated with markers of membrane damage, such as galectin-3, indicating a close interplay between autophagy and membrane repair processes (Morrison et al., 2023).

Galectins, a family of β-galactoside-binding proteins, have emerged as critical modulators of host–pathogen interactions in tuberculosis (Thurston et al., 2012). These proteins recognize glycosylation patterns on host and microbial surfaces, facilitating pathogen recognition and enhancing phagocytosis (Cummings et al., 2022). During M.tb infection, galectins such as galectin-3 and galectin-8 are recruited to damaged phagosomal membranes, where they act as danger sensors and promote selective autophagy (Bell et al., 2021; Morrison et al., 2023). Beyond their role in pathogen clearance, galectins influence granuloma formation, regulate cytokine and chemokine production, and help balance protective immunity with the risk of immunopathology (Morrison et al., 2023). At the molecular level, host factors such as the RNA-binding protein ZNFX1 have emerged as key regulators of autophagy during *M.tb* infection. ZNFX1 stabilizes mRNA encoding the AMPK catalytic subunit (Prkaa2), thereby sustaining AMPK-mediated autophagic flux. Loss of ZNFX1 impairs autophagy, reduces macrophage activation, and increases bacterial burden, underscoring its role in host resistance (Liu et al., 2024).

### 3.3 Modulation of host autophagy for survival

*M.tb* has developed a range of sophisticated strategies to evade host autophagy, a key innate immune mechanism that targets intracellular pathogens for lysosomal degradation. One major tactic involves the inhibition of autophagosome maturation and fusion with lysosomes, often mediated by the ESX-1 secretion system, which disrupts phagosomal membranes and facilitates cytosolic escape (Lerena and Colombo, 2011; Cardenal-Muñoz et al., 2017). *M.tb* also manipulates host signaling pathways to suppress autophagy, notably through the Eis protein, which activates the Akt/mTOR pathway, and PE/PPE family proteins like PE_PGRS47, which inhibit autophagy and antigen presentation (Duan et al., 2016). Additionally, *M.tb’s* lipid components—such as Mannose-capped lipoarabinomannan (ManLAM), phthiocerol dimycocerosates (DIMs) and sulfoglycolipids (SLs)—interfere with autophagy initiation and phagosomal acidification, further protecting the bacterium from degradation (Bah et al., 2020).

Beyond these direct mechanisms, *M.tb* disrupts LAP by impairing NADPH oxidase complex assembly via the CpsA protein and modulates host transcriptional and epigenetic landscapes to downregulate autophagy-related genes (Liu et al., 2014). It also exploits the host cytokine environment by promoting a Th2/M2 macrophage phenotype, which is less conducive to autophagy (Ding et al., 2024). Furthermore, *M.tb* interferes with PRR signaling, particularly TLR2 and TLR9, to prevent autophagy activation (Ding et al., 2024). These multifaceted evasion strategies enable *M.tb* to persist within macrophages, contributing to latent infection and disease progression. Understanding these mechanisms is crucial for developing host-directed therapies aimed at restoring autophagic function and enhancing bacterial clearance.

### 3.4 Autophagy and macrophage polarization

Autophagy plays a crucial role in the immune response to intracellular pathogens like *M.tb*, and its regulation is closely tied to Th1 and Th2 cytokines. IFN-γ, a Th1 cytokine, induces autophagy in macrophages, a process that involves the immunity-related GTPase Irgm1 in mice and its human ortholog IRGM (Naik et al., 2024). Knockdown of these genes impairs autophagosome formation and enhances mycobacterial survival. Although the exact mechanism remains unclear, autophagy appears to be essential for IFN-γ-induced phagosome maturation, as evidenced by the requirement of Beclin 1 (Dutta et al., 2012). TNF-α, another Th1 cytokine, also promotes autophagy and contributes to host defense by inducing apoptosis and enhancing antigen presentation (Kleinnijenhuis, et al., 2011a, b; Sharma et al., 2014). It modulates autophagy through multiple pathways, including JNK activation and Akt inhibition, and its effects are context-dependent, varying across cell types and signaling environments (Kleinnijenhuis, et al., 2011a, b).

In contrast, Th2 cytokines such as IL-4 and IL-13 inhibit autophagy. These cytokines signal through IL-4Rα-containing receptor complexes, activating the IRS-1/2 and STAT6 pathways. In macrophages, IL-4 and IL-13 suppress both starvation- and IFN-γ-induced autophagy (Harris et al., 2007). The inhibition of starvation-induced autophagy is mediated via the Akt pathway, while suppression of IFN-γ-induced autophagy is STAT6-dependent. This inhibition reduces phagosome maturation and facilitates the intracellular survival of mycobacteria (Harris et al., 2007; Deretic et al., 2009). These findings suggest that Th2 responses can counteract the protective autophagic mechanisms promoted by Th1 cytokines, potentially undermining host defense against *M.tb*.

Overall, the balance between Th1 and Th2 cytokines significantly influences autophagy in macrophages, impacting the outcome of mycobacterial infections. A Th1-dominant environment, as seen in active tuberculosis, supports autophagy and enhances bacterial clearance through both NOS2-dependent and -independent mechanisms (Alonso et al., 2007; Davis et al., 2007; Vandal et al., 2008). Autophagy not only aids in pathogen elimination but also contributes to antigen processing and presentation, reinforcing adaptive immunity. Conversely, Th2 cytokines, possibly induced by virulent mycobacterial strains, may inhibit autophagy in an autocrine manner, promoting bacterial persistence (Harris et al., 2007). A recent *in vivo* study in mice indicate that autophagy plays a limited role in directly controlling Mycobacterium tuberculosis within macrophages. Instead, autophagy has a more pronounced effect on the immune response. It significantly influences CD4^+^ T cell polarization, particularly the balance between Th1 and Th2 subsets. This modulation of T cell responses contributes to enhanced immune control of tuberculosis infection. Along with T cell polarization autophagy also influenced the recruitment of neutrophils and disease pathology. This interplay highlights autophagy as a critical immunological process modulated by cytokine signaling and central to the host-pathogen interaction in tuberculosis.

### 3.5 Salmonella

*Salmonella* is a genus of bacteria that causes foodborne illness known as salmonellosis. *Salmonella* can survive and replicate inside host cells, particularly macrophages, within a specialized compartment called the Salmonella-containing vacuole (SCV).

*Salmonella* actively invades host macrophages and triggers autophagy through multiple mechanisms, primarily mediated by LAP (Cemma et al., 2011). Using a zebrafish embryo model, researchers found that live *Salmonella* triggers LC3 recruitment to bacteria-containing compartments in macrophages, a process dependent on ATG5 but independent of the autophagy preinitiation complex (e.g., ATG13), indicating involvement of LAP. Key regulators of LAP, including Rubicon (RUBCN) and NADPH oxidase, are essential for both LC3 recruitment and activation of ROS within infected macrophages. Several *Salmonella* effector proteins contribute to autophagy induction, including L-asparaginase (which depletes L-asparagine and inhibits mTOR) (Torres et al., 2016), SipD (which facilitates invasion and autophagy signaling) (Hernandez et al., 2003), and β-barrel outer membrane proteins (β-OMPs), which act as pathogen-associated molecular patterns (PAMPs) and trigger autophagic responses (Hernandez et al., 2003).

The bacterium’s escape mechanisms are largely driven by virulence factors encoded by the SPI-2 pathogenicity island and associated plasmids. Effectors such as SsrB and SsaV disrupt the Sirt1/LKB1/AMPK axis, reactivating mTOR and suppressing autophagy (Ganesan et al., 2017). Meanwhile, SseF and SseG inhibit ULK1 recruitment by blocking Rab1A, thereby impairing autophagosome formation (Feng et al., 2018). SseL, a deubiquitinase, removes ubiquitin tags from *Salmonella*, preventing recognition by autophagy adaptors (Feng et al., 2018). These effectors collectively help maintain the integrity of the SCV and create a replicative niche shielded from host defenses.

Plasmid-encoded factors also contribute to autophagy evasion. The pR (ST98) plasmid in *Salmonella Typhi* and the spv locus in other pathogenic strains inhibit autophagy and promote intracellular survival. Notably, spvB disrupts actin polymerization and suppresses autophagy in macrophage cell lines (He et al., 2012). Additionally, *Salmonella* produces reactive persulfides like CysSSH and GSSH, which interfere with 8-nitro-cGMP signaling, a pathway essential for autophagy-mediated bacterial clearance in macrophages (He et al., 2012).

Host factors are also co-opted in this process. FAK is recruited to the SCV via SPI-2, where it activates the Akt-mTOR pathway, further suppressing autophagy. In FAK-deficient macrophages, autophagic clearance of *Salmonella* is enhanced, and bacterial survival is significantly reduced (He et al., 2012). This highlights how *Salmonella* manipulates both bacterial and host signaling pathways to evade autophagy and establish chronic infection.

### 3.6 Brucella

*Brucella* is a genus of Gram-negative bacteria that causes brucellosis. In humans, *Brucella* primarily resides in macrophages, where it survives and replicates inside specialized compartments, evading immune responses.

Recent research has deepened our understanding of how *Brucella* manipulates host autophagy and macrophage polarization to establish chronic infection and evade immune responses. After entering host cells, *Brucella* resides in *Brucella-*containing vacuoles (BCVs), which mature through interactions with endosomal and ER-derived compartments (Starr et al., 2008). This process is tightly regulated by the bacterium’s Type IV secretion system (T4SS), which delivers effectors that manipulate host pathways, including autophagy (Zhang et al., 2019). At later stages, *Brucella* transitions into autophagic vacuoles (aBCVs), exploiting the autophagy machinery to facilitate replication and cell-to-cell spread, while evading lysosomal degradation (Celli, 2015).

Autophagy also intersects with macrophage polarization, a process critical to the immune response. *Brucella* skews macrophages toward an M2 phenotype, which is associated with anti-inflammatory responses and tissue repair, thereby creating a permissive environment for bacterial persistence. Exosomal miR-let-7e-5p is significantly downregulated in brucellosis patients. This downregulation promotes M2 polarization via the Rictor/AKT1 signaling pathway. Overexpression of miR-let-7e-5p, conversely, enhances M1 polarization and suppresses M2, suggesting a potential therapeutic target to restore effective immune responses (Li et al., 2025). *Brucella* also exploits the NF-κB signaling pathway in regulating macrophage polarization during Brucella abortus infection. Initially, infection induces M1 polarization and pro-inflammatory cytokine production, but over time, this shifts toward M2 polarization, which correlates with increased bacterial survival. NF-κB was shown to regulate this switch by targeting the glutaminase gene (Gls), which influences macrophage metabolism and phenotype. Inhibiting NF-κB or Gls promotes M2 polarization and enhances intracellular *Brucella* survival, underscoring the importance of this pathway in host-pathogen dynamics (Zhao et al., 2023). Together, these findings illustrate how *Brucella* intricately manipulates autophagy and macrophage polarization to evade immune clearance and establish chronic infection.

### 3.7 Toxoplasma

*Toxoplasma gondii* is an obligate intracellular protozoan parasite that causes toxoplasmosis in humans and other warm-blooded animals and can cause chronic encephalitis (Anand et al., 2015b; Lutshumba et al., 2020; Anand et al., 2022)*. T. gondii* primarily resides in nucleated cells, especially macrophages, muscle cells, intestinal epithelial cells, and neurons. Inside these cells, it forms a parasitophorous vacuole where it survives and replicates without being destroyed by lysosomes. These host cells play an important role in the development of the parasite and various free radicle ions may inhibit or kill the parasite (Anand et al., 2016; Anand, 2024).

CD40-CD154 signaling plays a pivotal role in stimulating autophagy as a host defense mechanism against *T. gondii*. Engagement of CD40 by CD154 activates multiple autophagy-inducing pathways, including CaMKKβ–AMPK–ULK1, TNF-α–JNK–Beclin 1, and PKR, leading to autophagosome formation and lysosomal fusion with the parasitophorous vacuole (Subauste et al., 2007). One key mechanism is CD40 ligation, which has been shown to induce autophagic clearance of the parasite in murine and human macrophages. Upon CD40 activation, LC3 and lysosomal markers like LAMP1 and Rab7 are recruited to the parasitophorous vacuole (PV), suggesting fusion with endo-lysosomal compartments(Andrade et al., 2006). This process requires synergy with TNF-α and involves TRAF6-mediated signaling that enhances TNF-α production and activates autophagy via Beclin1 and ULK1 (Subauste et al., 2007; Subauste, 2009). Although the PV remains structurally intact during this process, the parasite is still targeted for degradation, indicating a unique autophagic route. This CD40-dependent mechanism is crucial for controlling *T. gondii* in both peripheral tissues and the central nervous system (Subauste, 2009).

*T. gondii*, however, has evolved strategies to evade autophagic clearance. It activates EGFR-Akt signaling in host cells, which prevents LC3 recruitment to the PV and blocks Beclin1- and Atg7-dependent autophagy. This evasion is mediated by parasite-derived microneme proteins MIC3 and MIC6, which contain EGF-like domains that stimulate host EGFR (Muniz-Feliciano et al., 2013). Additionally, a second evasion mechanism involves activation of the FAK-Src-EGFR-STAT3 pathway, which inhibits autophagosome formation (Muniz-Feliciano et al., 2013).*T. gondii* exploits the FAK-Src-STAT3 in tissues with lower EGFR expression as well by activating Src, which inhibits PTEN and recruits activated Akt to the parasitophorous vacuole, suppressing autophagy.These evasion strategies are active even in the absence of CD40 stimulation and highlight the parasite’s ability to manipulate host signaling to avoid destruction. Pharmacological inhibition of EGFR, such as with Gefitinib, has been shown to reduce parasite replication, suggesting potential therapeutic avenues (Corcino, 2019).

Another critical pathway for autophagic control of *T. gondii* involves IFN-γ-induced GTPases, particularly IRGs and GBPs, which disrupt the PV membrane and expose the parasite to the host cytoplasm (Corcino, 2019). Once exposed, the parasite can be targeted by non canonical autophagy, as evidenced by inability of LC3 vesicles harboring parasite to fuse with endosomes and lysosomes. This process has been observed in various cell types, including brain endothelial and retinal cells (Selleck et al., 2015). The IRG protein Irgm3 plays a key role in this response, localizing to autophagosomal membranes and facilitating parasite clearance (Zhao et al., 2009). These findings underscore the importance of both immune signaling and autophagy in controlling *T. gondii*, and they open new questions about how host cells detect and respond to vacuolar pathogens through coordinated autophagic and immune mechanisms.

Autophagy proteins play a crucial, non-canonical role in the immune control of *T. gondii*, particularly in murine cells. While early studies suggested that Atg5 restricted parasite replication, it became clear that this was not through classical autophagy, as PVs were not uniformly acidic and lacked consistent LAMP1 positivity. Instead, autophagy-related proteins such as Atg5, Atg7, Atg3, and Atg16L1 are essential for recruiting host immunity-related GTPases (IRGs) and guanylate-binding proteins (GBPs) to the PV membrane (PVM), facilitating its disruption (Khaminets et al., 2010; Zhao et al., 2023). These Atg proteins do not form isolation membranes but instead act as scaffolds for GTPase activation and targeting. The Atg12–Atg5–Atg16L1 complex appears to localize to the PVM via phosphoinositide-binding effectors, although the precise recruitment signals remain unclear (Fu et al., 2023). This recruitment is critical for initiating the immune response, as mis localization of these complexes leads to failed GTPase targeting and parasite persistence (Zhao et al., 2023).

In human cells, the autophagic response to *T. gondii* is more variable and cell-type dependent. Unlike mice, humans lack functional IFN-inducible IRGs, which may explain the absence of observed PVM rupture in human cells. However, humans do express IFNγ-inducible GBPs, such as hGBP1–5, which may or may not localize to the parasite in certain cell types like HAP1 and mesenchymal stromal cells (Kim et al., 2012; Johnston et al., 2016). In epithelial HeLa cells, autophagy proteins like Atg7 and Atg16L1 restrict parasite growth through a non-canonical mechanism that does not involve lysosomal fusion or PVM disruption (Selleck et al., 2015). Instead, LC3B and other autophagy-related membranes accumulate around the PV, suggesting a containment strategy. Ubiquitin tagging of the PV, along with recruitment of autophagy adaptors like p62 and NDP52, appears to be a common theme in human cells, although the exact mechanism of parasite restriction remains unresolved (Selleck et al., 2015).

The interplay between autophagy and *T. gondii* is further complicated by the parasite’s ability to exploit host autophagy for its own benefit, potentially using it as a nutrient source. Additionally, differences in parasite strain, host cell type, and immune status significantly influence the outcome of infection. For example, CD40 ligation has been shown to restore IFNγ and IL-12 production in immunodeficient patients, linking adaptive and innate immune responses (Séguin and Kasper, 1999). These findings underscore the complexity of autophagy-mediated control of *T. gondii* in the host.

### 3.8 Leishmania

*Leishmania* is a genus of protozoan parasites that cause leishmaniasis. In humans, *Leishmania* primarily resides in macrophages, where it survives and multiplies inside phagolysosomes and evading the immune system, show virulence and cause visceral or cutaneous leishmaniasis (Shoeran et al., 2025). Various natural and conventional drug therapies are available for the treatment of these parasites *in vitro* and *in vivo* (Kaur et al., 2021; Kim et al., 2023; Sharma et al., 2023; Chauhan et al., 2024; Kadayat et al., 2024).

Autophagy plays a pivotal role in host defense by degrading intracellular pathogens and regulating immune responses. However, *Leishmania* species have evolved sophisticated mechanisms to manipulate host autophagy for their own benefit. This manipulation is species-specific and temporally regulated, allowing the parasite to evade immune detection, acquire nutrients, and establish infection.

In the case of *L. donovani*, the parasite exhibits a biphasic modulation of host autophagy. Initially, it suppresses autophagy by activating the PI3K-Akt-mTOR signaling pathway, which inhibits classical autophagy. This suppression is marked by the accumulation of p62/SQSTM1, a ubiquitin-binding protein involved in autophagic cargo sequestration. After 24 hours, L*. donovani* switches to inducing autophagy via an mTOR-independent pathway. This shift is associated with reduced levels of IP3 and decreased activity of inositol monophosphatase (IMPase), disrupting the inositol signaling pathway. The delayed induction of autophagy is crucial for the parasite’s intracellular survival, allowing it to optimize nutrient acquisition from the host (Olivier et al., 1992; Thomas et al., 2018).

*L. major* displays a different strategy, inducing autophagy throughout its differentiation process in bone marrow-derived macrophages (BMDMs). This is evidenced by elevated levels of LC3B-II and a higher LC3B-II/LC3B-I ratio, indicating increased autophagosome formation. Key autophagy-related proteins such as ATG5, BNIP3, and ubiquitin are upregulated, supporting robust autophagic activity (Frank et al., 2015). Interestingly, this autophagy is not linked to mTOR inhibition; instead, hyperphosphorylation of mTOR and ribosomal protein S6 (RPS6) is observed. BNIP3, a protein involved in mTOR-independent autophagy, is significantly elevated, along with cathepsin E and HIF1A, which regulate autophagy and glycolytic gene expression. These changes facilitate the clearance of L. major amastigotes from host macrophages (Frank et al., 2015).

In contrast, *L. amazonensis* induces autophagy that correlates with increased parasite burden, particularly in BALB/c mice, which are more susceptible to infection (Pinheiro et al., 2009). Unlike *L. major*, autophagy in *L. amazonensis*-infected macrophages does not enhance nitric oxide (NO) production or alter arginase activity, suggesting a non-protective autophagic response. The parasite’s intracellular proliferation is dependent on ATG5-mediated autophagy, and essential autophagy proteins such as ATG8, ATG4.1, ATG5, and ATG12 are required for this process (Pinheiro et al., 2009).

*Leishmania* parasites themselves rely on autophagy for differentiation and virulence. The ATG8 conjugation system, supported by ATG5 and ATG12, is essential for autophagosome formation and phospholipid homeostasis (Veras et al., 2019). Disruption of ATG5 impairs ATG8 function, leading to mitochondrial dysfunction and reduced virulence. Parasites with functional autophagy elicit CD4+ T cell responses and reduce survival, while those with defective autophagy enhance T cell proliferation and infectivity (Crauwels et al., 2015).

## 4 M2 macrophage polarization and autophagy as immune evasion strategies

Macrophage polarization plays a dominant role in various infectious diseases and cancer (Anand et al., 2023). In parasitic and intracellular bacterial infections, the immune system activates macrophages via Th1 and Th2 pathways, which distinctly influence autophagy and pathogen control (Martinez and Gordon, 2014). The Th1 response, driven by IFN-γ and IL-12, induces classically activated M1 macrophages that are pro-inflammatory and effective at eliminating intracellular pathogens such as *Leishmania*, *M.tb*, *Salmonella*, and *Brucella(* Desmedt et al., 1998 *)*. These M1 macrophages enhance autophagy, a critical host defense mechanism that degrades intracellular microbes and promotes antigen presentation (Desmedt et al., 1998). In contrast, the Th2 response, mediated by IL-4 and IL-13, promotes alternatively activated M2 macrophages, which support tissue repair and suppress inflammation (Desmedt et al., 1998). While M2 macrophages are beneficial against extracellular parasites like helminths and aid granuloma formation in diseases like schistosomiasis, they inhibit autophagy, creating a niche for intracellular pathogens to thrive (Desmedt et al., 1998). Many pathogens exploit this Th2/M2 axis to evade immune clearance. Thus, the balance between Th1/Th2 responses, macrophage polarization, and autophagy is pivotal in determining infection outcomes and host susceptibility.

*Leishmania and Toxoplasma* are intracellular protozoan parasites that have evolved to manipulate host immune responses, particularly by skewing macrophage polarization toward the M2 phenotype state associated with tissue repair, immune suppression, and parasite tolerance (Anand et al., 2015a; Kong et al., 2015; Tomiotto-Pellissier et al., 2018). In *Leishmania* infections, especially with *L. infantum*, parasite burden correlates with increased arginase-1 (Arg1) activity and reduced inducible nitric oxide synthase (iNOS) expression, shifting macrophages away from a microbicidal M1 phenotype (Iniesta et al., 2002). This shift is further supported by the parasite’s mimicry of apoptotic cells through phosphatidylserine exposure, which engages CD36 on macrophages and activates PPARγ signaling, promoting M2 polarization (Gallardo-Soler et al., 2008). Additionally, *Leishmania*-infected dendritic cells accumulate neutral lipids, impairing antigen presentation and further dampening T cell responses (Gallardo-Soler et al., 2008).

Autophagy plays a dual role in this context. On one hand, several *Leishmania species* induce autophagy in host macrophages, likely to access nutrients and support intracellular survival. For example, *L. amazonensis* and *L. donovani* infections are associated with increased LC3 expression and autophagosome formation (Gallardo-Soler et al., 2008; Thomas et al., 2018). This autophagy induction may synergize with M2 polarization by promoting metabolic reprogramming and lipid droplet formation, which are hallmarks of M2 macrophages (Thomas et al., 2018). Moreover, autophagy can suppress antigen presentation and pro-inflammatory signaling, reinforcing the immunosuppressive environment that favors parasite persistence (Gan et al., 2023). In some cases, autophagy may even attenuate CD4+ T cell responses, as seen with LC3-positive membranes surrounding apoptotic *L. major*, further contributing to immune evasion (Jacquin and Apetoh, 2018; Na and Engwerda, 2024).

*Toxoplasma* similarly exploits M2 polarization through the secretion of virulence factors like ROP16, which directly phosphorylates STAT3 and STAT6, bypassing cytokine signaling and inducing M2-associated genes such as Arg1. This manipulation promotes an anti-inflammatory macrophage phenotype that supports parasite survival (Chen et al., 2020). Autophagy intersects with this process as well: *T. gondii* can induce or subvert autophagy depending on the host cell type and immune context. While autophagy can contribute to parasite clearance under certain conditions, *T. gondii* often hijacks autophagic pathways to avoid lysosomal degradation and acquire nutrients (Cheng et al., 2022). Together, M2 polarization and autophagy form a coordinated strategy by which these parasites evade immune destruction and establish chronic infections. Understanding this interplay offers promising avenues for therapeutic intervention aimed at reprogramming macrophage responses and restoring effective host immunity.

## 5 Discussion

The Akt-mTOR signaling axis is central to macrophage activation and polarization, influencing immune responses through its regulation of metabolism, cytokine production, and autophagy. mTORC1 and mTORC2, activated by distinct upstream signals, differentially modulate macrophage phenotypes—mTORC1 promotes pro-inflammatory M1 responses and autophagy, while mTORC2 supports anti-inflammatory M2 polarization and immune tolerance. This balance is critical in infectious diseases, where Th1-driven M1 macrophages enhance autophagy and pathogen clearance, particularly against intracellular bacteria and protozoa *like Mycobacterium, Salmonella, Leishmania*, and *Toxoplasma*. Conversely, Th2-mediated M2 polarization suppresses autophagy, creating a permissive niche for pathogen survival. Intracellular parasites such as *Leishmania* and *T. gondii* exploit this axis by inducing M2-associated genes (e.g., Arg1) and manipulating host signaling—Leishmania through apoptotic mimicry and PPARγ activation, and Toxoplasma via ROP16-mediated STAT3/6 phosphorylation. Both pathogens also modulate autophagy to support their intracellular persistence, either by inducing autophagosome formation for nutrient acquisition or by subverting lysosomal degradation. These strategies underscore the complex interplay between macrophage polarization, autophagy, and immune evasion, revealing potential therapeutic targets to reprogram macrophage responses and restore effective host immunity.

## 6 Future perspectives

Autophagy is regulated by a range of proteins and their inhibitors, which can either activate or suppress the process. Exploring how these modulators interact with intracellular pathogens—either alone or in combination with antiparasitic drugs—could yield valuable insights into the role of autophagy during infection.

While the role of autophagy in macrophages and its downstream effects on CD4^+^ T cell responses is well-established in the context of M.tb, where murine models have demonstrated a clear link between macrophage autophagy and T cell polarization, similar evidence for other pathogens remains incomplete. Current studies often address isolated aspects, such as the impact of autophagy inhibition on macrophage polarization or cytokine secretion, without integrating these findings into a broader immunological context.

To build a more comprehensive understanding, future research should leverage advanced technologies capable of simultaneously profiling immune cell subtypes and macrophage phenotypes in the context of intracellular infections. *In vivo* studies using autophagy-deficient mouse models, or pharmacological modulation of autophagy through specific inhibitors and activators, will be essential. These approaches can help delineate the complex interplay between autophagy, macrophage function, and adaptive immune responses, ultimately clarifying the immunomodulatory role of autophagy across a broader spectrum of pathogens.

Additionally, the role of autophagy as both a host defense mechanism and a tool exploited by pathogens like *Leishmania* and *Toxoplasma* warrants deeper investigation. Therapeutic strategies that restore autophagic flux while promoting M1 polarization could enhance pathogen clearance and limit chronic infection. Future experiments may involve the use of autophagic inhibitors to assess infection outcomes in these diseases and examine their correlation with macrophage polarization states. Advances in single-cell transcriptomics based immune profiling, metabolic profiling, and *in vivo* imaging will be instrumental in unraveling the spatial and temporal dynamics of macrophage plasticity in disease settings. Ultimately, integrating immune metabolic insights with host-pathogen interaction studies may yield novel immunomodulatory therapies that harness macrophage plasticity to improve outcomes in infectious and inflammatory diseases.

## 7 Open questions

How do specific autophagy modulators (e.g., rapamycin, chloroquine, Torin1/2) influence macrophage polarization in the context of different intramacrophage pathogens and influence outcomes?

Can modulating autophagy in macrophages influence their cytokine profile and influence CD4+T cell polarization?

Various autophagy-deficient mouse strains are available, each exhibiting a block at different regulatory points of the autophagy pathway. How might infection outcomes—such as cytokine profiles and CD4⁺ T cell polarization—differ between these autophagy-deficient strains and wild-type controls?

How does autophagy manipulation affect other cells of innate and adaptive immune response apart from cd4+ T cell polarization?

## Data Availability

The original contributions presented in the study are included in the article/supplementary material. Further inquiries can be directed to the corresponding author.
